# Medicine

**Published:** 1892-03

**Authors:** Wm. C. Krauss


### MEDICINE.

BY WM. C. KRAUSS, M. D.

### ICHTHYOL IN THE TREATMENT OF DYSPEPSIA AND CEPHALALGIAS.

DR. STOCQUART, of Brussels, in experimenting upon ichthyol, says that it will relieve the pains of dyspepsia and of neuralgias. It is more active than the bromide of potash, better supported by the the stomach, improves the appetite, and hastens digestion. The dose, as recommended, is 0.50 centigrams daily.—*Le Progrès Médical.*

---

### A CASE OF CHOREA CURED BY EXALGINE.

THE patient, a child of eight years, who had been afflicted for a month, was relieved from the very onset by exalgine, and cured in the space of eighteen days. Twenty centigrams were given daily for a week and 30 centigrams after this time.—Dr. Moncorvo in *Le Progrès Médical.*

---

### THE ACTION OF ICE AND ICE WATER IN DIPHTHERIA.

THE treatment of diphtheria as employed by Dr. Bleyne consists in the application of ice upon the neck, and the internal use of ice. If ice is not obtainable, water as cold as possible may be used instead. The author claims that cold destroys the bacillus of diphtheria.

---

### THE ACCUMULATION OF BROMIDE OF POTASH IN THE DIFFERENT TISSUES.

FÉRÉ and Herbert, of Paris, have recently made some interesting experiments on this point and transmitted their report to the Société de Biologie. An epileptic, aged 64, began December 18, 1890, with 5 grammes daily of potassium bromide, then took six grammes daily, and, beginning October 13, 1891, 7 grammes daily until October 20, 1891. On October 23d he died with pneumonia. The various tissues of the body were then carefully examined and found to contain, per 100 grammes, the following quantities of the salt: brain, 0.073; lungs, 0.082; liver, 0.104; spleen, 0.133; kidneys, 0.10; pancreas, 0.043; muscle, 0.062; cartilage, 0.041; bone, 0.087.

---

### MODIFICATIONS OF THE PULSE OF THE BRAIN AND FOREARM DURING THE ADMINISTRATION OF SULFONAL.

DR. SGOBBO FRANCESCO, of Naples, in making a series of observations, published in *Annali di Neurologia, Fac. II.*, 1891, came to the following conclusions:

1. That sulfonal is a good hypnotic.

2. That given in doses of 3 grammes it exhibits an action over the heart and blood-vessels, reinforcing the systole and increasing the vascular tone of the arteries. This action upon the vessels is not continuous, for after a certain time there is dilatation and a progressive loss of elasticity, beginning first in the vessels of the brain, then extending to the periphery. The alterations in the vessels stand in relation to the amount of the drug taken.

### INTUBATION IN RUSSIA.

JAKUBOWSKI'S results are as follows:

1. Primary diphtheria of the larynx. In fifty-nine cases, intubation was performed in thirty-two, with twenty-one recoveries— 65.6 per cent.

Tracheotomy was performed in twenty-seven cases, with two recoveries—7.4 per cent.

2. In five cases of secondary diphtheria, where intubation was performed, there resulted three recoveries—60 per cent.

From these results the author draws the following conclusions:

1. Intubation is an operation less dangerous and less difficult than tracheotomy.

2. The inconveniences of intubation are: (*a*) The patient should be under the constant charge of the physician, and (*b*) the introduction and removal of the tube provokes disagreeable symptoms.

3. Secondary pneumonias follow more often intubation than tracheotomy.

4. In croup limited to the larynx and to the superior part of the trachea intubation is the procedure necessary.

5. In a diffuse croupous inflammation of the larynx, trachea, and bronchial tubes, tracheotomy is the operation required.—*Bull. Gen. de Thèrapeutique*, December 15, 1891.

